# Primary Dysmenorrhea: Assessment and Treatment

**DOI:** 10.1055/s-0040-1712131

**Published:** 2020-06-19

**Authors:** Inês Guimarães, Ana Margarida Póvoa

**Affiliations:** 1 Faculty of Medicine, Universidade do Porto, Porto, Portugal; 2 Department of Gynecology, Unit of Reproductive Medicine, Centro Hospitalar Universitário de São João, Porto, Portugal

**Keywords:** dysmenorrhea, menstrual cycle, pain, NSAIDs, hormonal contraception, dismenorreia, ciclo menstrual, dor, AINEs, contracepção hormonal

## Abstract

Primary dysmenorrhea is defined as menstrual pain in the absence of pelvic disease. It is characterized by overproduction of prostaglandins by the endometrium, causing uterine hypercontractility that results in uterine muscle ischemia, hypoxia, and, subsequently, pain. It is the most common gynecological illness in women in their reproductive years and one of the most frequent causes of pelvic pain; however, it is underdiagnosed, undertreated, and even undervalued by women themselves, who accept it as part of the menstrual cycle. It has major implications for quality of life, such as limitation of daily activities and psychological stress, being one of the main causes of school and work absenteeism. Its diagnosis is essentially clinical, based on the clinical history and normal physical examination. It is important to exclude secondary causes of dysmenorrhea. The treatment may have different approaches (pharmacological, non-pharmacological and surgical), but the first line of treatment is the use of nonsteroidal anti-inflammatory drugs (NSAIDs), and, in cases of women who want contraception, the use of hormonal contraceptives. Alternative treatments, such as topical heat, lifestyle modification, transcutaneous electrical nerve stimulation, dietary supplements, acupuncture, and acupressure, may be an option in cases of conventional treatments' contraindication. Surgical treatment is only indicated in rare cases of women with severe dysmenorrhea refractory to treatment.

## Resumo

Dismenorreia primária é definida como dor menstrual na ausência de patologia pélvica. Caracteriza-se pelo excesso de produção de prostaglandinas pelo endométrio que provocam hipercontractilidade uterina, resultando em isquemia e hipoxia do músculo uterino e, subsequentemente, dor. É a patologia ginecológica mais comum em mulheres em idade fértil e uma das causas mais frequentes de dor pélvica; contudo, é subdiagnosticada, subtratada, e até desvalorizada pelas próprias mulheres, que a aceitam como parte do ciclo menstrual. A dismenorreia tem grandes implicações na qualidade de vida, como limitação das atividades diárias e estresse psicológico, sendo uma das principais causas de absentismo escolar e laboral. O seu diagnóstico é essencialmente clínico, baseando-se na história clínica e num exame físico sem alterações. É importante excluir causas secundárias de dismenorreia. O tratamento pode ter diferentes abordagens (farmacológica, não farmacológica e cirúrgica), sendo que a primeira linha de tratamento consiste na utilização de anti-inflamatórios não esteroides (AINEs) e, em casos de mulheres que desejem contracepção, no uso de anticoncepcionais hormonais. Tratamentos alternativos, como a utilização de calor tópico, modificação do estilo de vida, estimulação elétrica nervosa transcutânea, suplementos alimentares, acupuntura e acupressão, podem ser uma opção nos casos de contraindicação da utilização dos tratamentos convencionais. O tratamento cirúrgico apenas se encontra indicado em casos raros de mulheres com dismenorreia grave e refratária aos tratamentos.

## Introduction

Primary dysmenorrhea is defined as colic pain in the suprapubic region with irradiation to the lumbar and thighs that occurs before or during menstruation in the absence of pelvic illness. 1 2 3 4 Its first manifestation usually appears 6 months after menarche because it occurs only during ovulatory cycles. The pain typically lasts from 8 to 72 hours and is most severe on the 1 ^st^ and 2 ^nd^ days of menstruation, due to increased release of prostaglandins during this period. The symptoms are reproducible from one menstrual period to another. It is associated with nausea, vomiting, diarrhea, low back pain, migraines, dizziness, fatigue, insomnia, and rarely, syncope and hyperthermia. 1 2 3 4 5 6 This results of prostaglandins’ action on the smooth muscles of the stomach, intestine, and blood tissues. 7 Symptom severity is positively correlated with early menarche, increased duration and amount of menstrual flow. 8

## Epidemiology

Primary dysmenorrhea is the most common gynecological disease in menstruating women. 1 2 6 9 Its prevalence is more significant during the second and third decades of life and decreases with advancing age, unlike secondary dysmenorrhea. 9 Its prevalence is underestimated and difficult to determine because only a small number of women seek medical treatment. Contributing to this are the different definitions of dysmenorrhea that exist and the lack of standard methods for defining its severity. 1 2 9 According to a systematic review by the World Health Organization in 2006, the prevalence of dysmenorrhea in menstruating women is between 17 and 81%. Severe dysmenorrhea was identified in only 12 to 14% of cases. 10

### Impact on Quality of Life

Dysmenorrhea causes high rates of school and work absenteeism, as well as decreasing quality of life. 5 11

In a study conducted in Portugal, 8.1% of girls reported missing school or work due to menstrual pain, impacting daily activities in ∼ 65.7% of cases. Only 27.9% sought medical help. 12 It mainly affects academic performance in terms of concentration, sport, socialization, and school achievement. It also influences pain tolerance and causes sleep disturbance, daytime fatigue and drowsiness. 1 11 According to the National Sleep Foundation's Women and Sleep Poll, women report sleep disturbance during the 1 ^st^ days of menstruation, and 28% report that sleep was disturbed by menstrual pain. 13

### Differential Diagnosis

Before attributing to menstrual pain the diagnosis of primary dysmenorrhea, it is important to exclude other diseases. Secondary dysmenorrhea is associated with underlying pelvic disease. It is characterized by later onset of symptoms than primary dysmenorrhea, usually more than 2 years after menarche. It may be associated with other gynecological symptoms, such as abnormal uterine bleeding. Pain has different characteristics and may persist beyond catamenium. 1 6 9 Endometriosis is the most common cause and, therefore, the most important differential diagnosis. It is the illness that best mimics the symptoms of primary dysmenorrhea and is characterized by the presence of endometrial tissue in extrauterine locations. Adenomyosis is another cause of secondary dysmenorrhea and is defined as a benign invasion of the myometrium by endometrial tissue. 1 14 When menarche occurs associated with pain, flow obstruction should be suspected as in Müllerian anomalies: imperforate hymen, transverse vaginal septum, or vaginal agenesis. 2 6 8 15 Abortion and ectopic pregnancy may present with severe pelvic pain and bleeding and should be the primary suspect in sexually active adolescents and young women. 8 Pelvic inflammatory disease should be suspected in the presence of sexually transmitted infection's history or abnormal vaginal discharge associated with dyspareunia. 4 Non-gynecological disorders include: gastrointestinal causes (irritable bowel syndrome, inflammatory bowel disease, chronic constipation and lactose intolerance), genitourinary causes (cystitis, renal lithiasis) and psychogenic causes (trauma, history of sexual abuse). 2 9 15

## Diagnosis

The evaluation of menstrual pain should include a detailed clinical history and physical examination, and it is important to exclude pelvic disease. Clinical history should include: menstrual history (age at menarche, regularity, and duration of cycles, amount of flow, time between menarche and onset of dysmenorrhea), pain characterization (type, location, irradiation, associated symptoms, chronology), treatments used, family history of dysmenorrhea, sexual history, system review (gastrointestinal, genitourinary, musculoskeletal, and psychosocial). 2 15 Pelvic examination should be performed in sexually active adolescents, women with severe pain or activity limitation, and in cases that do not respond to first and second-line treatments. In non-sexually active adolescents with no history of a systemic disease but typical of primary dysmenorrhea, a pelvic examination is not necessary, but abdominal examination should be performed to exclude other diseases. 2 4 6 7 8 15 Pelvic examination consists of: inspection of the external genital area (important for determining sexual maturity, visualization of the hymen [signs of trauma]), speculum examination (allows assessment of anatomical diseases such as flow obstruction or the presence of vaginal discharge suggestive of infection), bimanual examination (allows the uterus to be evaluated for its mobility, size, texture, and presence of masses such as fibroids and it also allows uterosacral attachments and ligaments to be evaluated for masses and nodules suggestive of endometriosis). 14 In primary dysmenorrhea, the pelvic examination shows no changes. 4 When history and physical examination suggest pelvic disease, further investigation should be undertaken to determine the causes of secondary dysmenorrhea using transvaginal ultrasound, magnetic resonance imaging, and, possibly, laparoscopy. 2 4 6 7 8 14

## Pathophysiology

The etiology of primary dysmenorrhea is characterized by increased synthesis and release of prostaglandins, which cause hypercontractility of the myometrium, resulting in uterine muscle ischemia and hypoxia and, subsequently, pain. 1 3 5 11 This statement is supported by: similarity between symptoms of primary dysmenorrhea and prostaglandin-induced uterine contractility in labor or abortion; prostaglandins in the menstrual fluid of women with primary dysmenorrhea and by demonstrating the efficacy of cyclooxygenase (COX) inhibitors in pain relief by inhibiting prostaglandin synthesis. 3 9 Prostaglandins are synthesized through long-chain polyunsaturated fatty acids such as arachidonic acid, a common component of cell membrane phospholipids. Its synthesis is limited by the availability of free fatty acid precursors, which are regulated by cyclic adenosine monophosphate (cAMP). Thus, through the cAMP pathway, prostaglandin production can be stimulated by adrenaline, peptide, and steroid hormones, but also by mechanical stimuli and tissue trauma. 1

**Fig. 1:**
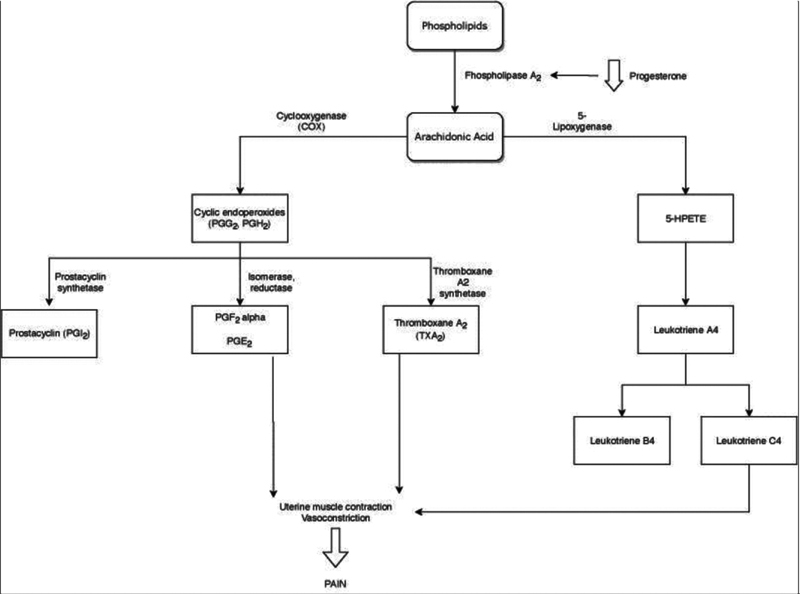
Prostaglandin synthesis pathway. Abbreviations: 5-HPETE, arachidonic acid 5-hydroperoxide; PGF _2a,_ prostaglandin F _2a_ ; PGE _2_ , prostaglandin E _2_ ; PGG _2_ , prostaglandin G _2_ ; PGH _2_ , prostaglandin H _2_ .

Arachidonic acid is derived from phospholipids through the lysosomal enzyme phospholipase A2 ( Fig. 1 ). The stability of lysosomal activity is regulated by several factors, one of which is progesterone. High progesterone levels stabilize lysosome activity. Decreased progesterone levels, which occur when the corpus luteum regresses in the late luteal phase, results in a decrease in this stabilizing effect on endometrial lysosomes, leading to the release of phospholipase A2 and hydrolysis of cell membrane phospholipids for arachidonic acid synthesis and of eicosatetraenoic acid. These compounds serve as precursors of the COX and lipoxygenase pathways. Thus, during menstruation, increased availability of arachidonic acid, intracellular destruction, and tissue trauma favor prostaglandin production. It was also possible to conclude that dysmenorrhea occurs only in ovulatory cycles. 1 3 5 11 15

There are 9 classes of prostaglandins, those implicated in the pathogenesis of primary dysmenorrhea are prostaglandin F(2α) PGF2α and prostaglandin E _2_ (PGE2). Prostaglandin F(2α) is more important in dysmenorrhea because it causes uterine vasoconstriction and contraction of the myometrium, while PGE2 causes either relaxation or contraction of the myometrium. Prostaglandins have several effects, including pain, inflammation, body temperature change, and sleep regulation. The larger the amount of prostaglandins, the greater the severity of menstrual pain and associated symptoms. 1 In addition to elevated prostaglandin levels, dysmenorrhea is also characterized by increased: uterine contractions, resting tone (> 10 mm Hg), active intrauterine pressure (> 120 mm Hg), frequency of contractions, and uncoordinated contractions. These anomalies lead to poor uterine perfusion, causing pain. 1 3

The role of prostanoids such as thromboxane A2, prostacyclins, and leukotrienes is not yet well established. Prostacyclins are a potent uterine vasodilator and relaxant and are thought to be decreased in dysmenorrhea. Leukotrienes, produced by the 5-lipoxygenase pathway, may be responsible for some forms of nonsteroidal anti-inflammatory drug (NSAID)-resistant dysmenorrhea. In women with dysmenorrhea, there is a greater number of leukotrienes in the menstrual fluid, especially C4 and D4 leukotrienes. Also, there are specific binding sites on myometrial cells for C4 leukotrienes, so leukotrienes are also thought to contribute to uterine hypercontractility. The involvement of vasopressin in the pathogenesis of dysmenorrhea is also controversial, but it is thought that high levels of circulation may produce dysrhythmic uterine contractions, leading to reduced uterine flow, hypoxia and, consequently, pain. 3 9 11 Recent evidence has shown that nitric oxide may also be involved, because it was found to be responsible for uterine quiescence during pregnancy. 11

Some studies have shown that women with dysmenorrhea have hypersensitivity to pain, but there is still no consensus on the subject. 1 It is thought that, in these cases, there is variation in the way pain is processed; the peripheral nociceptive information generated by the sexual organs during menstruation is amplified, causing increased excitability of the somatovisceral convergent neurons in the spinal cord and, consequently, increased pain perception. 16 17 Changes in brain structure were also observed in women with dysmenorrhea, such as lower gray matter volume in brain regions involved in pain transmission and sensory processing, and a larger volume of gray matter in regions involved in pain modeling and regulation of endocrine functions. These changes support the amplification of pain facilitation. 18

### Risk Factors

There are two types of risk factors for primary dysmenorrhea: non-modifiable and behavioral. Non-modifiable risk factors include: family history of dysmenorrhea, age under 20 years (symptoms are more pronounced during adolescence), menarche before age 12 (due to early establishment of ovulatory cycles), menstrual flow lasting longer than 7 days and nuliparity. 1 2 7 14 15 19 The association between multiparity and decreased risk of dysmenorrhea can be explained by several assumptions such as: lower release of prostaglandins by the endometrium after term delivery, neuronal degeneration that occurs in the uterus after a term delivery, and decreased uterine norepinephrine in the third trimester of pregnancy. 20 Behavioral risk factors include: body mass index (BMI) < 20 or > 30, low intake of omega 3 (fish), smoking (nicotine induces vasoconstriction), caffeine consumption (also induces vasoconstriction), and psychosocial symptoms such as depression and anxiety. Also, a stressful relationship with the parents may favor primary dysmenorrhea. 21 It is important to identify these behavioral factors as they are subject to intervention. 1 2 7 14 15 19 Stress inhibits the release of luteinizing and follicle-stimulating hormones, which leads to impaired follicular development with alteration in progesterone synthesis and release that influences prostaglandin activity. Besides, stress-related hormones, such as adrenaline and cortisol, also, influence prostaglandin synthesis and myometrial binding. 20 Some studies have shown an association between the occurrence of primary dysmenorrhea and other conditions that cause chronic pain, such as irritable bowel syndrome, migraines, and fibromyalgia. Women with primary dysmenorrhea are twice more likely to develop irritable bowel syndrome than women without dysmenorrhea. In addition, this condition may exacerbate symptoms of other diseases with the increased pain sensitivity. 22

### Treatment

**Fig. 2:**
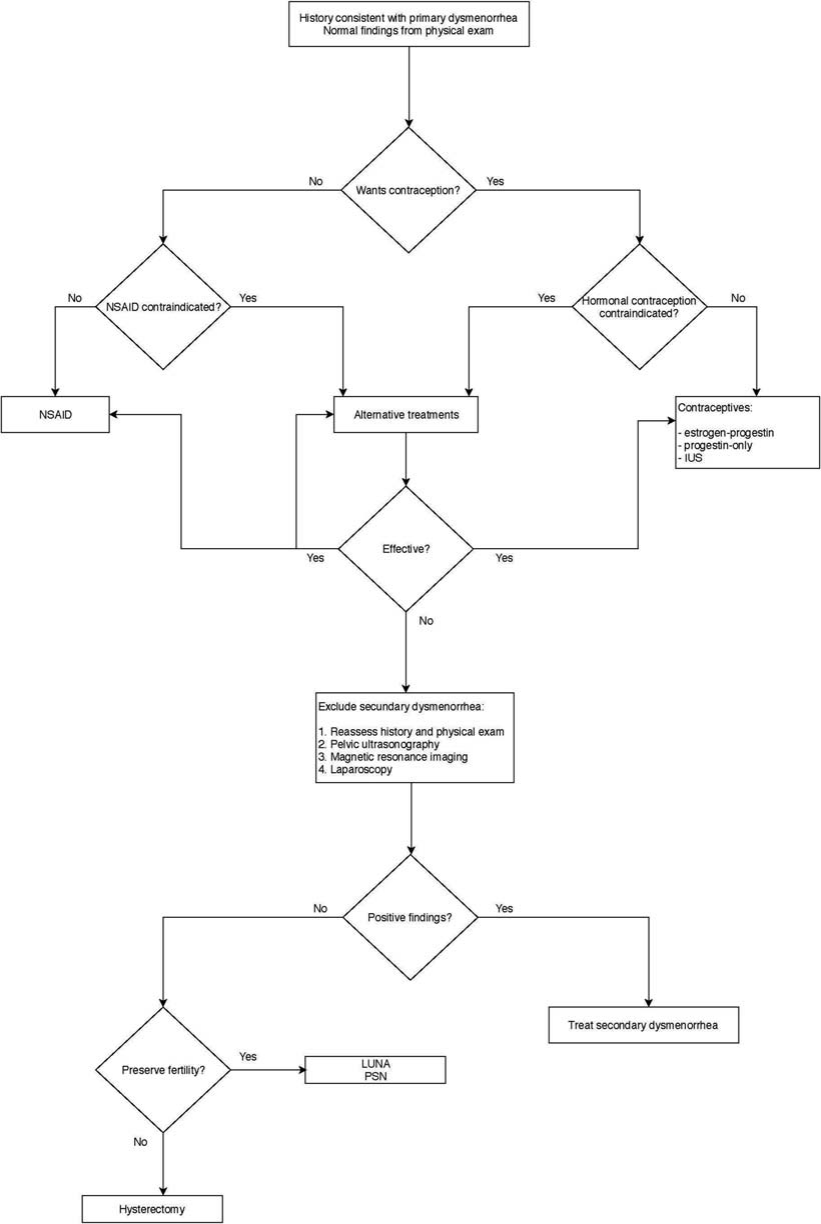
Flowchart on the treatment of dysmenorrhea. Abbreviations: NSAIDs, non-steroidal anti-inflammatory; IUS-intrauterine system; LUNA, laparoscopic uterosacral nerve ablation; PSN, presacral neurectomy.

The goal of the treatment is to provide adequate relief from menstrual pain. General measures for pain control include educating the patient about the physiology of menstruation and the pathophysiology of menstrual pain, reassurance and support. 9 23 There are three types of treatment for primary dysmenorrhea control: pharmacological, non-pharmacological, and surgical, with pharmacological treatment being the most effective ( Fig. 2 ). 3

#### Pharmacological

**NSAIDs:** These are considered the first line of treatment. They act by inhibiting COX, which results in decreased prostaglandin production and, consequently, decreased prostaglandin concentration in menstrual fluid, decreased uterine contractility, and menstrual volume. Its adverse effects are uncommon and well tolerated but mainly consist of gastrointestinal symptoms such as nausea, vomiting, and heartburn. Other less common adverse effects include nephrotoxicity, hepatotoxicity, hematological abnormalities, bronchospasm, fluid retention, and edema. There is still little evidence on which NSAID is more effective or safer, but, currently, the most widely used ones are ibuprofen, naproxen, mefenamic acid, and ketoprofen. 3 6 9 14 15 They are most effective when they start before the onset of symptoms and need to be continued for 3 days. 2 7 Cyclooxygenase II inhibitors are more specific and are less likely to induce duodenal ulcers. Because they cause serious adverse effects, they are no longer used for the treatment of primary dysmenorrhea. 3 8 15 23 24

### Hormonal Contraceptives

- Combined oral contraceptive (estrogen-progestin): This is the second line of treatment, acting to suppress ovulation and endometrial growth by causing a decrease in menstrual volume and prostaglandin secretion, with a subsequent decrease in intrauterine pressure and uterine contractility. 2 3 8 11 Continuous use is more effective than cyclic use because it decreases the frequency of menstrual periods leading to a decrease in dysmenorrhea. 2 9 They are effective in both primary and secondary dysmenorrhea, so their use should be initiated without waiting for the definitive diagnosis. 2 15
- Progestin-only methods: These drugs may be effective in treatment, as the progestin component is responsible for inducing endometrial atrophy, leading to pain relief, but has not yet been studied as well as combined oral contraceptives. 23 Progestin-only pill (desogestrel) decreases menstrual flow and ∼ 10% of women get amenorrheic. It can be used as an alternative to combined oral contraceptives, causing fewer adverse effects. 2 23 25 Long-acting injectable medroxyprogesterone acetate causes amenorrhea in 50% of women due to endometrial atrophy; however, due to effects on bone mineral density, it is not used currently. 2 7 8 9 23 The intrauterine levonorgestrel-releasing device (IUS) has been shown to reduce menstrual flow and endometrial thickness, reducing pain. 1 7 9 23 The non-hormonal intrauterine copper device is associated with increased menstrual flow and pain and is, therefore, not used. 9
- Transdermal and vaginal contraceptives: their use has not been well studied in primary dysmenorrhea but the effects of estrogen-progestin contraceptives on the endometrium are similar regardless of their methods of administration and should not affect their effectiveness. 23 26
- Subdermal implant with etonogestrel release: allows contraception for 3 years. Several studies have shown an improvement in symptoms of dysmenorrhea after use. 8

### Others

- Tocolytics: Since primary dysmenorrhea is caused by uterine hypercontractility, these drugs, by blocking contractility, maybe effective in treatment. Thus, nitric oxide, nitroglycerine and calcium channel blockers are being investigated as potential drugs for the treatment of dysmenorrhea, but they are not yet used for it. 23 Transdermal nitroglycerin is less effective than NSAIDs and has more adverse effects, such as migraines; therefore, due to their low tolerability, they are not used for treatment. 3 7 27 Calcium channel blockers such as nifedipine inhibit myometrium contractility by blocking calcium entry into cells of the smooth muscle, decreasing the pain. They are associated with several adverse effects: transient facial flushing, increased heart rate, palpitations, and migraines. 3 23 28
- Vitamins: Vitamin E relieves primary dysmenorrhea because it suppresses phospholipase A2 and COX activity, thus inhibiting prostaglandin production and promoting prostacyclin action, with consequent vasodilation and muscle relaxation. 3 5 Vitamin B1 supplementation can improve dysmenorrhea by reversing common symptoms of B1 deficiency, such as muscle cramps, fatigue, and decreased pain tolerance. 5
- Omega 3: Dysmenorrhea is associated with diets rich in omega 6 and low in omega 3 fatty acids. Increased incorporation of omega 3 into membrane phospholipids through ingestion of fish oil leads to lower production of prostaglandins and leukotrienes. Adverse effects include: nausea and acne exacerbation. 3 5 7

#### Non-Pharmacological

**Lifestyle changes:** The role of arachidonic acid as a precursor to prostaglandin production led to the thought of the role of diet in controlling dysmenorrhea. Thus, changes in diet such as low-fat diet, ingestion of beans, seeds, fruits and vegetables allow a decrease in arachidonic acid production. Besides, physical exercise seems to decrease the symptoms of dysmenorrhea. A general benefit of exercise is reported in women younger than 25 years of age who exercise at least 45 to 60 minutes, 3 times a week. 29 Implementing a healthy lifestyle with proper nutrition, exercise, smoking cessation, and low alcohol consumption alleviates the symptoms of dysmenorrhea and minimizes its discomfort and inconvenience. 2 7 9 Some studies show lack of evidence of any dietary supplement to reduce dysmenorrhea and also the lack of safety data of these products. 30

**Topical heat:** consists of the direct application of heat in the suprapubic region; it is an effective and low-cost natural method. 2 3 9

**Transcutaneous Electrical Nerve Stimulation (TENS):** It allows pain relief through two mechanisms. The first is to raise the threshold for pain signals caused by uterine hypoxia and hypercontractility by sending a series of afferent impulses through the large-diameter sensory fibers of the same nerve root, resulting in less pain perception. The second mechanism is the stimulation of endorphin release by the peripheral nerves and spinal cord thus providing another partial pain attenuation pathway. 3 5 It may be an alternative in women with contraindications to the use of NSAIDs and its effects. Adverse effects include: muscle stiffness, migraines, nausea, skin redness or burn. 2 5

**Acupuncture and acupressure:** The mechanism of acupuncture involves stimulation of nerve fibers and receptors in a complex interaction with endorphins and serotonins. 5 9 Some evidence suggests their use as a treatment, but it is still insufficient for their recommendation, and further studies are needed to prove their effectiveness. 31 32 They may be an alternative in women not interested in pharmacological treatment. 2 3

#### Surgical

Only indicated in rare cases of women with severe and treatment-refractory dysmenorrhea, requiring re-evaluation of the diagnosis and investigation of secondary causes; 33 however, there are very few studies on this topic and the ones that exist are old reports that have not been replicated enough to safely recommend these methods.

**Laparoscopic uterosacral nerve ablation (LUNA):** involves the transection of afferent pain fibers in the uterosacral ligaments. 9 33

**Presacral neurectomy (PSN):** direct transection of nerve fibers in the pelvis. 9 33

**Hysterectomy:** this method can be considered as the last resort in cases refractory to conventional therapy and in which laparoscopy reveals normal anatomy and absence of deep infiltration by endometriosis. 2 7

## Conclusion

Primary dysmenorrhea is one of the most common illnesses in women of childbearing age. When severe, it may interfere with the activities of daily living and may lead to school and work absenteeism. Its diagnosis is based on a characteristic clinical history, normal physical examination, and absence of pelvic disease. Its management consists of patient education, reassurance, and support. Pharmacological treatment is the most effective and includes NSAIDs or hormonal contraceptives for women who do not wish to become pregnant. Women who do not respond to treatment after 3 months of use should be investigated for suspected secondary dysmenorrhea. Primary dysmenorrhea is also one of the most underdiagnosed diseases, so clinicians should suspect the diagnosis in a timely manner and provide appropriate treatment.
